# Coffee and risk of death from hepatocellular carcinoma in a large cohort study in Japan

**DOI:** 10.1038/sj.bjc.6602737

**Published:** 2005-08-09

**Authors:** Y Kurozawa, I Ogimoto, A Shibata, T Nose, T Yoshimura, H Suzuki, R Sakata, Y Fujita, S Ichikawa, N Iwai, A Tamakoshi

**Affiliations:** 1 Department of Social Medicine, Division of Health Administration and Promotion, Faculty of Medicine, Tottori University, Nishimachi 86, Yonago 683-8503, Japan; 2 Department of Public Health, Kurume University School of Medicine, Kurume 830-0011, Japan; 3 Fukuoka Institute of Health and Environmental Sciences, Dazaihu 818-0135, Japan; 4 Department of Public Health, Niigata University School of Medicine, Niigata 951-8510, Japan; 5 Chugoku Occupational Health Association, Fukuyama 721-0942, Japan; 6 Department of Preventive Medicine/Biostatistics and Medical decision Making, Nagoya University Graduate School of Medicine, Nagoya 466-8550, Japan

**Keywords:** coffee, hazard ratio, hepatocellular carcinoma, Japan Collaborative Cohort Study (JACC Study)

## Abstract

We examined the relation between coffee drinking and hepatocellular carcinoma (HCC) mortality in the Japan Collaborative Cohort Study for Evaluation of Cancer Risk (JACC Study). In total, 110 688 cohort members (46 399 male and 64 289 female subjects) aged 40–79 years were grouped by coffee intake into three categories: one or more cups per day, less than one cup per day and non-coffee drinkers. Cox proportional hazards model by SAS was used to obtain hazard ratio of HCC mortality for each coffee consumption categories. The hazard ratios were adjusted for age, gender, educational status, history of diabetes and liver diseases, smoking habits and alcohol. The hazard ratio of death due to HCC for drinkers of one and more cups of coffee per day, compared with non-coffee drinkers, was 0.50 (95% confidence interval 0.31–0.79), and the ratio for drinkers of less than one cup per day was 0.83 (95% confidence interval 0.54–1.25). Our data confirmed an inverse association between coffee consumption and HCC mortality.

Hepatocellular carcinoma (HCC) has a high incidence in Africa and Asia where Hepatitis B virus (HBV) or Hepatitis C virus (HCV) infection are major risk factors (Tabor, 1998). Heavy alcohol consumption (La Vecchia *et al*, 1988; Tanaka *et al*, 1992) and dietary aflatoxins (Bulatao-Jayme *et al*, 1982; Yu *et al*, 1999) increase the risk of HCC, while diabetes (La Vecchia *et al*, 1997), smoking (La Vecchia *et al*, 1988; Yu *et al*, 1999) and low education level (La Vecchia *et al*, 1988) are also reported risk factors.

Coffee drinking has been inversely related to the risk of liver cirrhosis in several studies (Klatsky and Armstrong, 1992; Klatsky *et al*, 1993; Corrao *et al*, 1994, 2001; La Vecchia *et al*, 1998; Gallus *et al*, 2002a), although no significant relation was found in two case–control studies from Italy (La Vecchia *et al*, 1989) and Greek (Kuper *et al*, 2000). To investigate further an association between coffee drinking and HCC mortality, we analysed data from the Japan Collaborative Cohort Study for Evaluation of Cancer Risk sponsored by Monbusho (JACC Study).

## SUBJECT AND METHODS

### Subjects

Subjects were 110 792 cohort members (46 465 males and 64 327 females) aged 40–79 years from JACC Study, the design of which has been previously described (Ohno *et al*, 2001). The subjects were followed up from 1988–1990 until the end of 1999. Residential and survival status was confirmed by searching in roster of residents for moving out or death, and in death certificate for cause and date of death under the permission from the Director-General of Prime Minister's Office.

End point for the present study was death from HCC, coded as C22.0 in International Classification of Diseases and Related Health Problems, 10th Revision (ICD-10). Subjects with HCC at baseline or died from HCC within 2 years after registration in the study were excluded from the analysis. Subjects coded C22.9 (hepatic malignancy not otherwise specified) were also excluded from the analysis. The total number of subjects was 110 688 (46 399 males and 64 289 females). The subjects who died of HCC during the observation periods were 287 male and 114 female subjects.

### Questionnaire and data correction

After obtaining the informed consent to participate the study, subjects were interviewed or completed questionnaire. A self-administered questionnaires for the survey included past and family history, health condition and lifestyle habits such as smoking, alcohol and non-alcohol beverages, diet, physical exercise, occupation and others. On the questionnaire, habitual coffee consumption was queried by the question’ Do you drink coffee? ‘The response sets was: ‘almost everyday’; ‘3–4 cups per week’; ‘1–2 cups per week’; ‘1–2cups per month’; ‘scarcely any’. Those who answered ‘almost everyday’ were asked to report the number of cups of coffee per day. Study participants were grouped into three groups as follows: one or more cups per day, less than one cup per day and 0 cup per day (non coffee drinkers). Drinkers of less than one cup per day included those of ‘3–4 cups per week’, ‘1–2 cups per week’ and ‘1–2cups per month’.

Each data set was transformed into the format of the JACC Study standard questionnaire, submitted to central office. Integrated data were tested in distribution and logical accuracy by working group for data clean up. More detailed process was described elsewhere (Shibata *et al*, 2003).

### Data analysis

SAS version 8.2. software (SAS institute, Cary, NC, USA) was used for the statistical analysis. To examine the association between the potential confounding factors and coffee consumption, we calculated the age-adjusted proportions and mean values for each factor at each coffee level. Cox proportional hazards model by SAS PHREG with strata statement (difference of collaborating institutes) was used to obtain hazard ratio (HR) of HCC mortality for each coffee consumption categories. Multiple logistic regression analysis was used to analyse the trends in adjusted means and proportion. *P*-values for trends in the Cox analysis were calculated by assigning a median value of cups per day to each categories. Chi-squared test was used to assess differences between proportions. All results were considered to be significant at the 5% critical level.

This study was approved by the Ethics Committee of the Kurume University School of Medicine.

## RESULTS

Table 1 shows the age distribution and the age-adjusted rates of lifestyle characteristics by coffee consumption categories. Persons who consumed larger amount of coffee tended to be younger. Coffee consumption was associated with education level, smoking and alcohol habits.

Table 2 shows HRs of death from HCC according to coffee consumption. The HR of death due to HCC for drinkers of one and more cups of coffee per day, compared with non-coffee drinkers, was 0.50 (95% confidence interval 0.31–0.79), and the ratio for drinkers of less than one cup per day was 0.83 (95% confidence interval 0.54–1.25). The HR of death due to HCC was significantly decreased among drinkers of one or more cups of coffee per day compared with non-coffee drinkers in men, but not in women. When the analysis was restricted to subjects with a history of liver disease, the HR of death due to HCC significantly decreased for drinkers of one and more cups of coffee per day compared with non-coffee drinkers. The decrease in risk was not significant when the analysis was restricted to subjects who did not report history of liver diseases.

## DISCUSSION

No consistent association emerged between coffee consumption and the risk of HCC. Recently, Gallus *et al* (2002b) reanalysed the Italian (La Vecchia *et al*, 1989, 1998) and Greek (Kuper *et al*, 2000) case–control studies of HCC and found an inverse association between coffee drinking and HCC risk. Japanese workers (Inoue *et al*, 2005) reported from 10-year follow-up data of the Japan Public Health Center-based Prospective (JPHC) Study that habitual coffee drinking may be associated with reduced risk of HCC. Further studies are warranted to determine whether the inhibitory effect applies to other population. We performed multivariate analysis of an association between coffee drinking and HCC mortality controlling the potential confounding factors age, gender, history of liver diseases, alcohol, smoking, education level, and diabetes in the JACC study. Our data showed an inverse association between coffee consumption and HCC mortality. Thus, the inhibitory effect of coffee drinking has been observed in the two different large-scale cohort studies.

In Japan, approximately 80% of HCC cases are associated with HCV (Yoshizawa, 2002). The multivariate HR of HCC mortality among coffee drinkers significantly decreased among those with a history of liver disease, but did not among those without such a history. The effect of coffee drinking on hepatocellular cartinogenesis may be associated with inhibiting the progression from hepatitis to cirrhosis or from cirrhosis to HCC. Another possible explanation is that persons might reduce coffee intake because of symptoms related to impaired caffeine clearance (Hasegawa *et al*, 1989) of poor liver function or nonspecific medical advice. Coffee consumption, however, was not significantly associated with history of liver diseases at baseline survey. Animal data have suggested an inhibitory effect of coffee on HCC (Tanaka *et al*, 1990). The inhibitory effect of coffee may be related to inhibition on the process of nitrosamine formation or hepatocarcinogenesis by nitrosamines. As for a possible effect of caffeine, green tea which contains caffeine is not significantly associated with HCC mortality in univariate analysis of the JACC Study (Kurozawa *et al*, 2004). The absence of serum markers for HCV or HBV infection is a major limitation, although HRs were adjusted by history of liver disease. Although the mechanism of the inhibitory effect is not clear, coffee drinking may have a real effect in reducing HCC mortality.

## MEMBER LIST OF THE JACC STUDY GROUP

The present investigators involved, with the co-authorship of this paper, in the JACC Study and their affiliations are as follows: Dr Akiko Tamakoshi (present chairman of the study group), Nagoya University Graduate School of Medicine; Dr Mitsuru Mori, Sapporo Medical University School of Medicine; Dr Yutaka Motohashi, Akita University School of Medicine; Dr Ichiro Tsuji, Tohoku University Graduate School of Medicine; Dr Yosikazu Nakamura, Jichi Medical School; Dr Hiroyasu Iso, Institute of Community Medicine, University of Tsukuba; Dr Haruo Mikami, Chiba Cancer Center; Dr Yutaka Inaba, Juntendo University School of Medicine; Dr Yoshiharu Hoshiyama, University of Human Arts and Sciences; Dr Hiroshi Suzuki, Niigata University School of Medicine; Dr Hiroyuki Shimizu, Gifu University School of Medicine; Dr Hideaki Toyoshima, Nagoya University Graduate School of Medicine; Dr Kenji Wakai, Aichi Cancer Center Research Institute; Dr Shinkan Tokudome, Nagoya City University Graduate School of Medical Sciences; Dr Yoshinori Ito, Fujita Health University School of Health Sciences; Dr Shuji Hashimoto, Fujita Health University School of Medicine; Dr Shogo Kikuchi, Aichi Medical University School of Medicine; Dr Akio Koizumi, Graduate School of Medicine and Faculty of Medicine, Kyoto University; Dr Takashi Kawamura, Kyoto University Center for Student Health; Dr Yoshiyuki Watanabe, Kyoto Prefectural University of Medicine Graduate School of Medical Science; Dr Tsuneharu Miki, Graduate School of Medical Science, Kyoto Prefectural University of Medicine; Dr Chigusa Date, Faculty of Human Environmental Sciences, Mukogawa Women's University; Dr Kiyomi Sakata, Wakayama Medical University; Dr Takayuki Nose, Tottori University Faculty of Medicine; Dr Norihiko Hayakawa, Research Institute for Radiation Biology and Medicine, Hiroshima University; Dr Takesumi Yoshimura, Fukuoka Institute of Health and Environmental Sciences; Dr Akira Shibata, Kurume University School of Medicine; Dr Naoyuki Okamoto, Kanagawa Cancer Center; Dr Hideo Shio, Moriyama Municipal Hospital; Dr Yoshiyuki Ohno, Asahi Rosai Hospital; Dr Tomoyuki Kitagawa, Cancer Institute of the Japanese Foundation for Cancer Research; Dr Toshio Kuroki, Gifu University; and Dr Kazuo Tajima, Aichi Cancer Center Research Institute.

## Figures and Tables

**Table 1:** Potential confounding factors acccording to coffee consumption categories by gender

	**Men**				**Women**			
	**Non-drinkers**	**<a cup day^−1^**	**⩾a cup day^−1^**	**Trend *P***	**Non-drinkers**	**<a cup day^−1^**	**⩾a cup day^−1^**	**Trend *P***
No	12 461	9490	21 828		19 346	12 710	28 767	
*Age (%)*								
40–49 years	16.0	23.3	32.1		13.8	24.7	31.1	
50–59 years	30.6	31.4	29.5		31.3	33.8	29.9	
60–69 years	36.0	31.0	26.0		36.8	29.4	26.9	
70–79 years	17.4	14.3	12.4		18.1	12.1	12.1	
								
Education^a^(%)	11.0	12.1	23.7	<0.001	7.4	8.1	14.3	<0.001
								
*Smoking habits*								
Currently smokes(%)	45.2	48.7	55.9	<0.001	3.7	4.0	7.3	<0.001
Smoked in the past(%)	30.5	29.4	25.7	0.295	1.7	2.0	2.0	0.354
								
*Alcohol habits*								
Currently drinks (%)	78.3	75.5	72.1	<0.001	17.9	26.4	26.5	<0.001
Drank in the past (%)	8.3	6.5	6.3	0.099	1.8	1.6	1.8	0.457
								
Liver disease (%)	9.9	8.9	7.4	0.845	6.6	6.8	5.3	0.109
								
Diabetes mellitus (%)	9.0	7.7	6.5	0.251	6	4.1	4.2	0.492

a Education: age at final graduation of 18 years old or more.

**Table 2:** Hazard ratios (HR) and 95% confidence interval (CI) for the association of HCC mortality with coffee consumption by gender and history of liver diseases

	**All subjects**			**Men**			**Women**			**Subjects with history of liver diseases**			**Subjects without history of liver diseases**		
	**No of subjects**	**No of cases**	**HR (95% CI)^a^**	**No of subjects**	**No of cases**	**HR (95% CI)^b^**	**No of subjects**	**No of cases**	**HR (95% CI)^a^**	**No of subjects**	**No of cases**	**HR (95% CI)^c^**	**No of subjects**	**No of cases**	**HR (95% CI)^c^**
Non-drinkers	24 556	103	1	9516	66	1	15 040	37	1	1991	62	1	22 565	41	1
<a cup day^−1^	15 259	57	0.83 (0.54–1.25)	6505	41	0.91 (0.57–1.45)	8754	16	0.64 (0.27–1.51)	1150	35	0.94 (0.53–1.66)	14 109	22	0.79 (0.44–1.41)
⩾a cup day^−1^	44 151	98	0.50 (0.31–0.79)	19 158	71	0.49 (0.28–0.85)	24 993	27	0.51 (0.20–1.31)	2686	54	0.44 (022–0.88)	41 465	44	0.61 (0.32–1.16)
*P* for trend			0.007			0.007			0.141			0.028			0.113

a Adjusted for age, gender, educational status, history of diabetes and liver diseases, smoking and alcohol habits.

b Adjusted for age, educational status, history of diabetes and liver diseases, smoking and alcohol habits.

c Adjusted for age, gender, educational status, history of diabetes smoking and alcohol habits.
